# A Sunken Ship of the Desert at the River Danube in Tulln, Austria

**DOI:** 10.1371/journal.pone.0121235

**Published:** 2015-04-01

**Authors:** Alfred Galik, Elmira Mohandesan, Gerhard Forstenpointner, Ute Maria Scholz, Emily Ruiz, Martin Krenn, Pamela Burger

**Affiliations:** 1 Institute of Anatomy, Histology and Embryology, Vetmeduni, Vienna, Austria; 2 Institute of Population Genetics, Vetmeduni, Vienna, Austria; 3 Institute of Prehistory and Historical Archaeology, Univ. Vienna, Austria; 4 Federal Office for the Protection of Monuments Vienna, Austria; Vrije Universiteit Brussel, BELGIUM

## Abstract

Rescue excavations recovered a skeleton that resurrect the contemporary dramatic history of Austria in the 17^th^ century as troops besieged Vienna in the second Osmanic-Habsburg war. Unique for Central Europe is the evidence of a completely preserved camel skeleton uncovered in a large refuse pit. The male individual of slender stature indicates a few but characteristic pathological changes revealing not a beast of burden but probably a valuable riding animal. Anatomical and morphometrical analyses suggest a hybrid confirmed by the ancient DNA analyses resulting in the presence of a dromedary in the maternal and a Bactrian camel in the paternal line.

## Introduction

**Fig 1:**
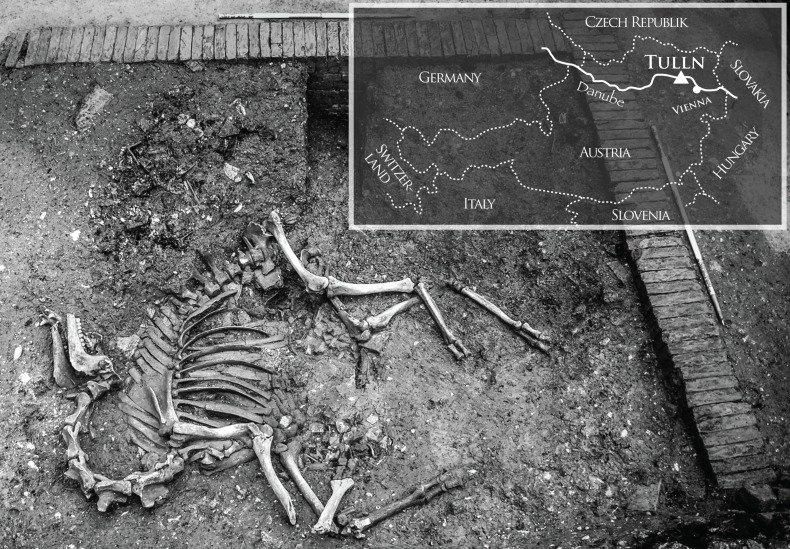
In situ view of the camel in the cellar. Inserted map indicates the geographical position of the town Tulln in Austria with a triangle.

Prior to the construction of a shopping center, excavations rescued the archaeological heritage of Tulln (Fig. 1) in Lower Austria in 2006 and 2007. The permission for the excavation was issued by the Federal Office for the Protection of Monuments in Vienna. Significant finds revealed large parts of the medieval and the early modern town including several building plots of an urban district [1]. An ensemble of buildings was specified as the historically mentioned tavern called “*Auf der Rossmühle*” near the large market place [2]. An abandoned cellar associated with this plot yielded the most extraordinary find of a complete skeleton originating from a large animal. The partly excavated skeleton was suspected to be a large horse or cattle, until the first author undoubtedly identified it as the remains of a camel (Fig. 1). The skeleton was found in a typical post-mortal posture with its neck bent backwards and drawn-up extremities [3].

Archaeological camel finds in Central Europe are not as unusual as one may expect covering a chronological span from Roman period till early modern age [4]. Yet, to this date only isolated bones or partly preserved camel skeletons have been found, e.g. at the Amphitheatre Viminacium in Serbia [5]. Its chronological position and the completeness of the skeleton highlights the uniqueness of the Tulln record. It is the first complete camel skeleton found in Central Europe and Central European territories under the control of the Ottoman Empire [6,7,8,9,10,11,12], apart from the complete skeleton of a dromedary recovered from the sediments of the Theodosius harbor on the European part of Istanbul [13]. In this study we combine archaeological as well as complementary morphologic and molecular genetic analyses to unravel the mystery of the sunken ship of the desert at the river Danube in Tulln.

## Archaeological and Historical Context

The backfill of the cellar yielded masses of domestic refuse like animal bones and ceramics (e.g. plates, pans and flagons), pieces of a tiled stove and enameled pipe bowls which date the filling in the early modern period, although the main part of the excavated material is not completely studied yet. Metal finds allowed a narrow and high resolution dating of the context. A coin—a so called “*Rechenpfenning”* depicts the countenance of King Louis XIV of France was dated from 1643 to 1715. A medicinal bottle made of lead contained the remedy “*Theriacum*” produced in the chemist’s shop “*Apotheke zur Goldenen Krone*” (approximately 1628/1665) in Vienna. These two exceptional finds place the filling containing the skeleton to the late 17^th^ century. Tulln was affected by floods of the Danube and plagues such as the Black Death, which drastically reduced the urban development [14] and a reduction of the inhabited area occurred due to conflagration and demolition of houses in the early 17^th^ century [2]. Two building plots got new owners at the end of the century around the 1690, certainly the time when the cellar was backfilled, offering enough space to bury such a big cadaver in the center of the town.

In summer 1683, Ottoman troops tried to reach Vienna and combed the region south of the Danube. The surrounding of Tulln was besieged by the army division, but the town itself never was conquered [14]. On the contrary, written sources describe a peaceful surrender of two prisoners of war. The Ottoman commander released the imperial ambassador and his secretary in Tulln in August 1683 and obviously, as proven by written sources, the Ottoman troops were in contact with the inhabitants of Tulln [14].

## Material and Methods

### Taphonomy, preservation, morphometry and statistics

**Fig 2:**
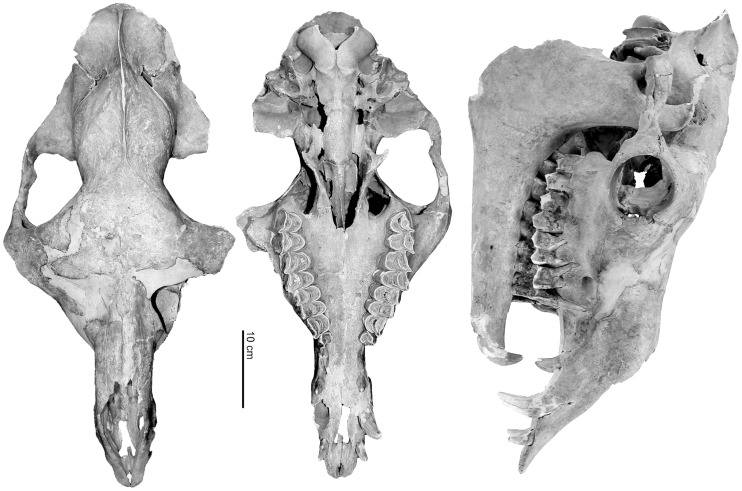
Reconstructed Tulu cranium of the Tulln specimen.

The freshly unearthed and moist bones were rather fragile and especially the skull, ribs and vertebrae suffered. The skull was one of the last elements excavated and broke into many fragments and small splinters but most of them were recovered and allowed a reconstruction of a large part of the head (Fig. 2). Both mandibles were more or less completely preserved but miss their foremost parts. However, most of the axial and the perpendicular skeleton were recovered and nearly all elements are present (Table 1). Only small bones, like small carpals, tarsals, sesamoids and several distal phalanges were lost.

**Table 1:** Quantification of recovered skeletal elements of the Tulln specimen.

anatomical element	right	left	unpaired indet	total	anatomical element	right	left	unpaired indet	total
calva			1	1	costa	6	12	10	18
mandibula	1	1		2	cartilago costalis				25
scapula	1	1		2	coxa				`(1)
humerus	1	1		2	femur	1	1		2
antebrachium	1	1		2	patella	1	1		2
os carpi radiale		1		1	tibia	1	1		2
os carpi intermedium		1		1	talus	1	1		2
os carpi accessorium		1		1	calcaneus	1	1		2
os carpale secundum		1		1	os tarsi centrale	1			1
os carpale tertium		1		1	os tarsale secundum	1	1		2
os carpale quartum		1		1	os tarsale quartum	1	1		2
metacarpus	1	1		2	metatarsus	1	1		2
phalanges proximales anteriores periphaer	1	1		2	phalanges proximales posteriores periphaer	1	1		2
phalanges proximales anteriores medial	1	1		2	phalanges proximales posteriores medial	1	1		2
phalanges mediae anteriores peripher	1	1		2	phalanges mediae posteriores peripher	1	1		2
phalanges mediae anteriores medial	1	1		2	phalanges mediae posteriores medial	1	1		2
vertebrae cervicales			7	7	phalanges distales				3
vertebrae thoracales			12	12	total	28	39	37	122
vertebrae lumbales			7	7					

Nevertheless, most of the skeletal material was in extraordinary good condition, except for the pelvis, which was severely fragmented. The bones show neither cut- or butchery marks nor traces of carnivore or rodent gnawing. Reconstruction and morphological investigations were performed at the Institute of Anatomy, Histology and Embryology at the Veterinary Medicine University Vienna. The species identification was attempted by comparisons with the reference collection at the Institute and according to literature [4,15,16,17,18]. Measurements (S1 Table, S2 Table, S3 Table) were taken according to von den Driesch [19], Steiger [17] and Pigière and Henrotay [4]. Comparative metrical data obtained from Steiger’s dissertation [17] was used in bivariate- as well as multivariate canonical discriminant analysis to predict the species of the Tulln specimen by measurements from skeletal elements [20]. This unique historical skeleton offered the opportunity to identify the species and to examine not only the individual’s stature but its age, sex and pathological changes in a detailed diagnostic investigation. The camel skeleton (specimen number: Tulln EKZ 2007, Fnr. 2645, SE 6684, 2007.02.07) is stored in the Depot of the Federal Office for the Protection of Monuments in Mauerbach, Lower Austria, Austria and all necessary permits were issued by the Federal Office for the Protection of Monuments, which complied with all relevant regulations.

### Sample Preparation and DNA Extraction

The historic sample of the Tulln camel specimen was prepared in a dedicated ancient DNA (aDNA) laboratory at the Paleogenetic Core Facility, ArchaeoBioCenter, LMU Munich), following a range of standard contamination precautions. All steps (bone cutting, surface removing, DNA extraction) were carried out in separate rooms. DNA was extracted from bone material following the protocols described in Rohland and Hofreiter [21] and Rohland et al. [22]. The authentication criteria for aDNA studies, such as multiple independent PCR amplification and parallel extraction / PCR controls were performed.

### PCR Amplification of mtDNA and Nuclear Markers

To genetically confirm the ancestry of the camel sample from Tulln, both the maternally inherited mtDNA and the nuclear DNA (inherited from both parents) were recovered. Initially, 532 bp of the mtDNA control region (nt 15345–15877) were amplified in 14 overlapping fragments (S4 Table) to detect the maternal origin. In addition, 11 nuclear regions (125 bp) including diagnostic SNPs differentiating between dromedary and Bactrian camels [23] were screened in the historic specimen as well as in four control individuals (dromedary, Bactrian camel, F_1_ hybrid, F_1_ backcross; Table 2).

**Table 2:** Diagnostic single nucleotide polymorphisms (SNPs) to differentiate hybrids between dromedary and Bactrian camel.

			HP206	HP288	HP379	HP405	HP429	HP458	HP501	HP633	HP264	HP900	HP168
Sample	Species	Location	(G|C)	(A|T)	(A|G)	(C|G)	(C|A)	(C|T)	(T|A)	(C|G)	(C|T)	(C|G)	(C|T)
Drom814	C.dromedarius	Sudan	GG	AA	AA	CC	CC	CC	TT	CC	CC	CC	TT
DC158	C.bactrianus	Austria	CC	TT	GG	GG	AA	TT	AA	GG	TT	GG	CC
Hyb55	F1 backcross	Kazakhstan	CC	TT	GA	GG	AA	CT	AA	GG	TT	GG	CC
Hyb56	F1 hybrid	Kazakhstan	GC	TT	GA	GC	AC	CT	TA	GC	TT	GC	CT
Tulln	F1 hybrid	Austria	GC	TA	GA	GC	AC	CT	TA	GC	CT	GC	CT

The PCR amplification was carried out in 20μl volume containing 1X PCR buffer (Invitrogen), 4mM MgCl2 (Invitrogen), 1 mg/ml BSA (Invitrogen), 250 μM mix dNTPs (Invitrogen), 1.5 μM for each primer (Invitrogen), 0.5 U of AmpliTaq Gold (Invitrogen) and 5μl DNA template. The PCR mixes were amplified using an iCycler Thermal cycler (Bio-RAD) outside the ancient DNA lab. The amplification program consisted of initial denaturation at 94°C for 9 min followed by 60 cycles consists of 94°C for 20 sec, primer specific annealing temperature (55°C- 59°C) for 30 sec, 72°C for 30 sec and a final extension of 72°C for 4 min. The successful PCR products were purified using the QIAquick PCR purification kit (Qiagen) and sequenced in both directions on ABI 3730 XL-Analyzer (Eurofins MWG GmbH, Ebersberg, Germany). Each sequence position was determined from two independent PCR amplifications (Forward and Reverse) to avoid sequence errors caused by template damage [24]. The mitochondrial sequences from the Tulln individual were aligned with the dromedary mtDNA reference sequence (NC009849), using CodonCode Aligner v.3.7.1.2 (Codon Code Corporation, USA). To identify the maternal origin of the camel specimen, the obtained mtDNA sequence was compared to the NCBI nucleotide database sequences, using the BLAST tool (http://blast.ncbi.nlm.nih.gov/Blast.cgi) with default blastn parameters. The SNP genotyping was based on the observance of homozygous or heterozygous alleles for each locus. DNA aliquots of the modern camel samples were retrieved from the database at the Institute of Population Genetics (Vetmeduni Vienna, Austria, P. Burger, pers. communication). All samples were collected either non-invasively (i.e., hair) or during routine veterinary controls and did not involve endangered or protected species.

## Results

### Age and Sex

All cranial sutures were solidly fused (Fig. 2) as well as the late fusing epiphyses of the long bones. Most of the *apophyses* of the *tiberositates spinosae* remained unfused at the thoracic part of the spinal column. The cervical and the anterior part of the thoracic vertebrae indicate clearly visible unfused epiphyses of the anterior and the posterior extremities. From the 10^th^ thoracic vertebra onwards the *extremitates craniales* as well as the *extremitates caudales* appear solidly fused like in all lumbar vertebrae. As these vertebral apo- andepiphyses fuse very late they indicate, like the dentition—all permanent teeth are in regularly use—definitely an adult individual probably older than seven years [25]. Although fragmentarily preserved the pubis offers some hints for the sex of the Tulln specimen. A t*uberculum pubicum dorsale* and-*ventrale* is present but the typical female *pecten ossis pubis* is missing. The *corpus pubis* appears to be wide. The *eminentia iliopubica* is weakly developed while the cranial margin of the corpus of the pubis is smooth and unusually indistinctly structured for an individual of this age. However, the massive canines in the upper- and the lower jaw supports the classification as a male individual [15,25,26].

### Species identification and size

Significant parts of the scull were reconstructed in order to show its original form. The scull appears elongated with a long facial part and a slender snout as observed in dromedaries (Fig. 2). Although rather slender, the relation of the facial and cranial part of the scull resembles more a Bactrian camel than a dromedary [15,26,27,28].

To start with the postcranial elements, both rather fragmented scapulae reveal weak *tuberositas spina scapulae*. The divided *tuberculum supraglenoidale* of the left scapula indicates a *processus coracoideus* like in the Bactrian camel while the right shoulder blade shows a bulgy protruding *processus coracoideus* like in dromedary [17]. The canonical discriminant analysis correctly predicts about 84% of the comparative material and categorizes both scapulae of the Tulln specimen as Bactrian camel (Table 3).

**Table 3:** Descriptive of the canonical discriminance analyses of Bactrian camel- and dromedary bones.

		predicted group membership									
		bactrian	dromedary	n	Eigen-value	kanonical correlation	Wilks-Lambda	Chi-Quadrat	df	significance	measurements
Scapula	bactrian	92,90%	7,10%	14	1,843	0,805	0,352	18,807	6	0,005	HS, LD, SLC, GLP
	dromedary	27,30%	72,70%	11							LG, BG
	Tulln	2									
Humerus	bactrian	100%		17	7,096	0,936	0,124	46,009	6	0,000	GL, GLC, BC, KD
	dromedary		100%	11							HT, BT
	Tulln	2									
Os antebrachii	bactrian	100%		17	14,018	0,966	0,067	58,249	9	0,000	GL, Bp, BFp, SD
	dromedary		100%	13							Bd, BFd, BrFd
	Tulln	1	1								BuFd
Metacarpus	bactrian	100%		16	11,554	0,959	0,08	55,661	8	0,000	GL, Bp, SD, Bd
	dromedary		100%	12							BTm, TTm, BTl
	Tulln	2									TTl
Phalanx	bactrian	85,70%	14,30%	14	1,967	0,815	0,336	20,173	5	0,001	GL, Bp, Dp, SD, Bd
proximalis	dromedary	22,20%	77,80%	9							
anterior	Tulln	4									
Phalanx	bactrian	85,70%	14,30%	14	1,65	0,789	0,377	15,108	5	0,01	GL, Bp, Dp, SD, Bd
medialis	dromedary	16,70%	83,30%	6							
anterior		3	1								
Femur	bactrian	100%		16	3,639	0,886	0,216	30,692	6	0,000	GL, Bp, Dc, SD, Bd
	dromedary	9,10%	90,90%	11							BCm
	Tulln	1	1								
Tibia	bactrian	100%		16	13,104	0,964	0,071	58,221	6	0,000	GL, Bp, SD, Bd, Dd
	dromedary		100%	12							BFOm
	Tulln	2									
Metatarsus	bactrian	100%		16	9,806	0,953	0,093	52,361	8	0,000	GL, Bp, SD, Bd, BTm
	dromedary		100%	12							TTm, BTl, TTl
	Tulln	2									
Phalanx	bactrian	100,00%		14	2,817	0,859	0,262	23,44	5	0,000	GL, Bp, Dp, SD, Bd
proximalis	dromedary	22,20%	77,80%	9							
posterior	Tulln	4									
Phalanx	bactrian	100,00%		14	2,291	0,834	0,304	18,464	5	0,002	GL, Bp, Dp, SD, Bd
medialis	dromedary	16,70%	83,30%	6							
posterior	Tulln	4									

The proximal joint of the humerus shows a distinct transverse groove between the bases of the *tuberculum minus* and the *tuberculum intermedium*. This and the huge size of the *Caput humeri* indicates a Bactrican camel while the *crista epicondyli lateralis* resembles that of a dromedary [17]. By means of metrical dimensions the canonical discriminant analysis predicts all humeri correctly and classifies the Tulln specimen as a Bactrian camel (Table 3).

In cranial view the descending lateral part of the *fovea capitis* radii of the *ossa antebrachii* is divided by an incision like in Bactrian camels and the lateral as well as the medial tuber is less pronounced like in Bactrian camels [17]. Although the metrical characterization allows a 100% correct prediction of both species the canonical discriminant analysis classifies one *antebrachium* of the Tulln specimen as dromedary and the other as Bactrian camel (Table 3).

The *fovea capitis femoris* appears to be deep like in dromedary but without accentuated edges The *trochanter major* is weak like in dromedary but appears indented at its cranial side like in Bactrian camel. The *trochanter minor* is rather small and pointed resembling dromedaries [17]. Most of the femora of both species are correctly predicted (78%) by the canonical discriminant analysis. However, one femur of the Tulln specimen is classified as dromedary and the other as Bactrian camel (Table 3). According to Steiger [17] the shallow proximal and distal depressions of the *patellae* indicates a Bactrian camel.

The height and width of the *eminentia intercondylaris* of the tibia resembles a dromedary while the form of the *condylus lateralis* and the *area intercondylaris caudalis* and the narrow *margo cranialis* resemble the Bactrian camel [17]. The canonical discriminant analysis predicts all tibiae correctly and classifies the Tulln specimens as Bactrian camel (Table 3).

**Fig 3:**
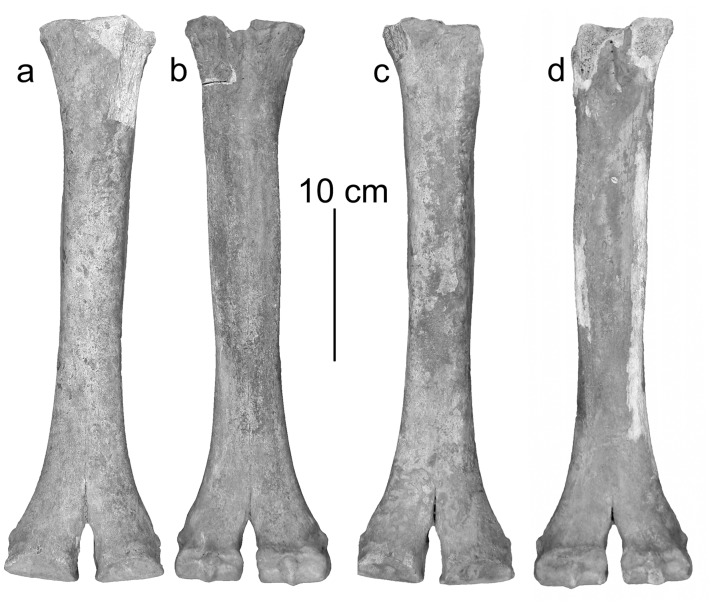
Left metacarpus: a) in dorsal- and b) in palmar view, right metacarpus: c) in dorsal- and d) in palmar view.

**Fig 4:**
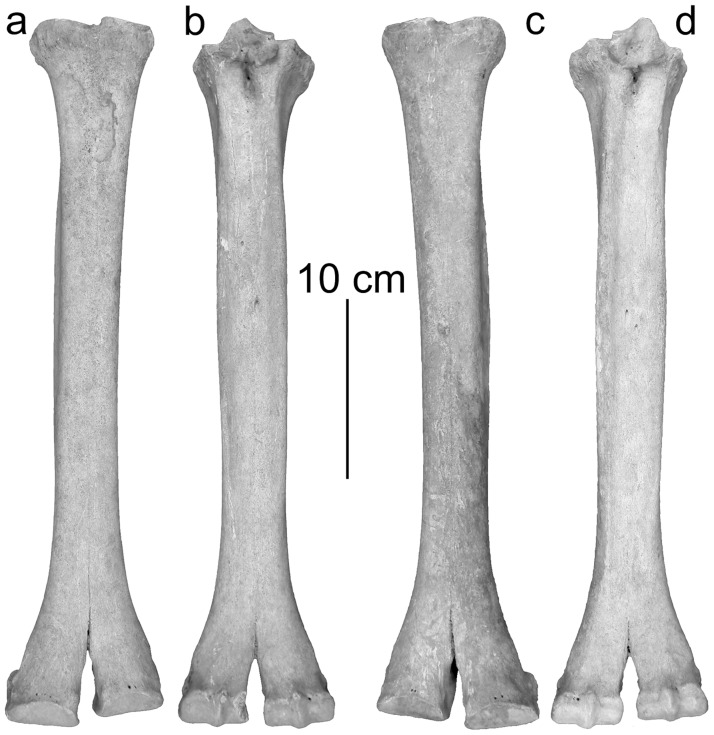
Left metatarsus: a) in dorsal- and b) in plantar view, right metatarsus: c) in dorsal- and d) in plantar view.

In dorsal view the proximal joint of the metacarpus and metatarsus shows a less developed difference in height at the step from the third to the fourth metapodial resembling dromedary [17] (Fig. 3, Fig. 4). However, the highly significantly discriminating canonical discriminant functions classify metacarpals and metatarsals of the Tulln specimen as being Bactrian camel (Table 3).

**Fig 5:**
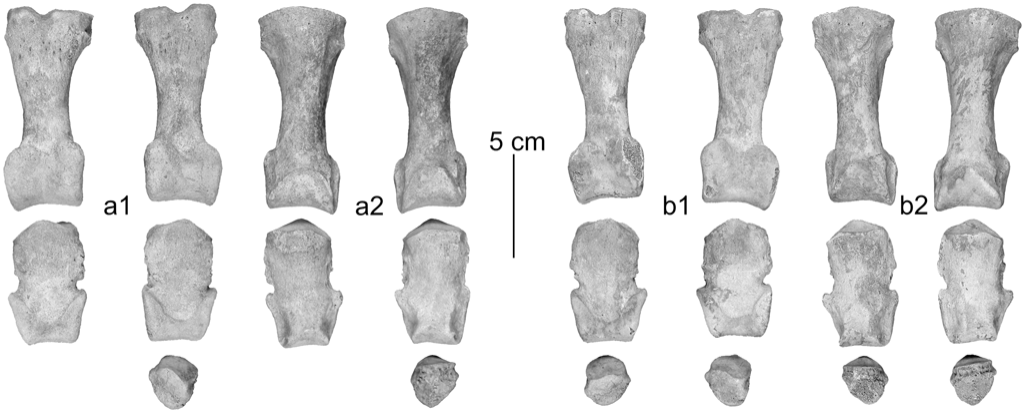
a) Left anterior phalanges in 1) palmar- and 2) dorsal view, b) right anterior phalanges in 1) palmar- and 2) dorsal view.

**Fig 6:**
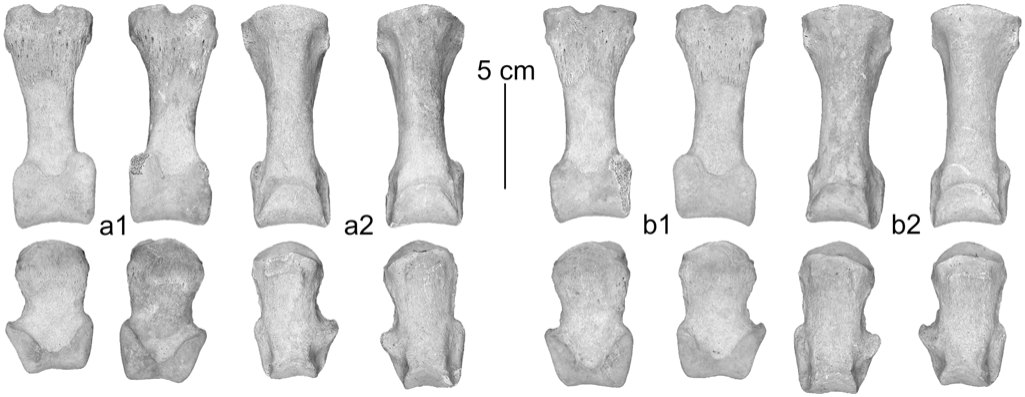
a) Left posterior phalanges in 1) plantar- and 2) dorsal view, b) right posterior phalanges in 1) plantar- and 2) dorsal view.

According to Studer and Schneider [18] and Pigière and Henrotay [4] the proximal phalanges show a well-developed lip at the palmar margin of the distal articular surface, forming a clear border against the corpus in dromedary. This characteristic does not clearly appear on the phalanges of the Tulln specimen (Fig. 5, Fig. 6). The difference of robustness is expressed by the canonical discriminant analyses. 82% of the anterior- and 91% of the posterior proximal phalanges are correctly predicted and all of the Tulln camel were classified as Bactrian camel. 85% of the anterior- and 95% of the posterior medial phalanges are correctly predicted. One of the anterior phalanges of the Tulln specimen is classified as dromedary and all four posterior medium phalanges are classified as Bactrian camel (Table 3).

Following Steiger [17] morphological features of the first two cervical vertebrae are distinctive to distinguish both species. The formation of the *foramina alaria* and the *foramina vertebralia lateralia* of the atlas indicate a dromedary. Nevertheless, the left side of the axis forms like in dromedary while the other side shows no pillar between the *foramen transversarium* and the *foramen vertebrale* like in Bactrian camel and the form of the *processus transversi* indicate an intermediate position between both species.

**Fig 7:**
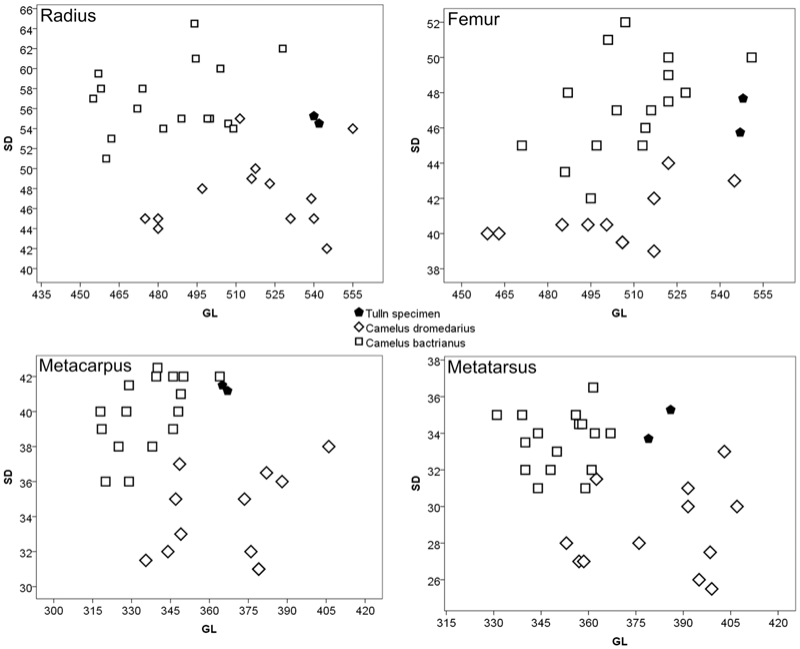
Bivariate scatterplots of radius, metacarpus, femur and metatarsus indicating size and robustness of the Tulln specimen by greatest length (GL) and smallest diameter of the shaft (SD) in comparison to data obtained from Steiger [17].

Compared to Steiger’s [17] data the long bones of the Tulln specimen indicate a rather large individual. Nevertheless the robustness of radius, femur and metatarsus, considering the relations of measured bone lengths and widths, takes an intermediate position between both species (Fig. 7).

### Molecular genetic analysis

For the identification of the species status of the *Camelus* specimen we compared the obtained mtDNA consensus sequence with the complete nucleotide database of NCBI. The BLASTn search results show significant hits to the dromedary mtDNA control region (E-value 0.0). These results confirm the dromedary as maternal ancestor of the specimen from Tulln. In addition to the maternally inherited mtDNA, we screened eleven nuclear SNPs, fixed in dromedary and Bactrian camels [23]. The eleven diagnostic SNPs present the homozygous pattern in the pure dromedary (Drom814) and Bactrian camel (DC158) controls, respectively (Table 2). The F_1_ hybrid (Hyb56; dromedary female x Bactrian male) expresses the expected heterozygous profile for all but two loci (HP264, HP288). The F_1_ backcross (Hyb55) shows heterozygosity for only two loci (18%) (HP379, HP458) remaining homozygous for the Bactrian allele in the other loci. Based on this set of eleven diagnostic SNPs, the morphologically ambiguous camel from Tulln represents the heterozygous pattern of an F_1_ hybrid in all positions, confirming the inheritance of both, dromedary and Bactrian camel variants.

### Pathologic changes

**Fig 8:**
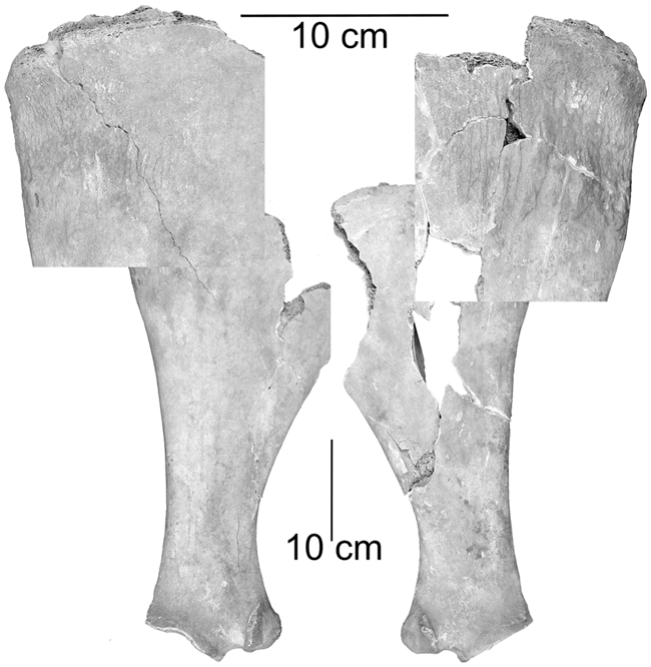
Left and right scapulae, enlarged view of both anguli caudales depict symmetrically formed pathological bone depositions on the medial side.

**Fig 9:**
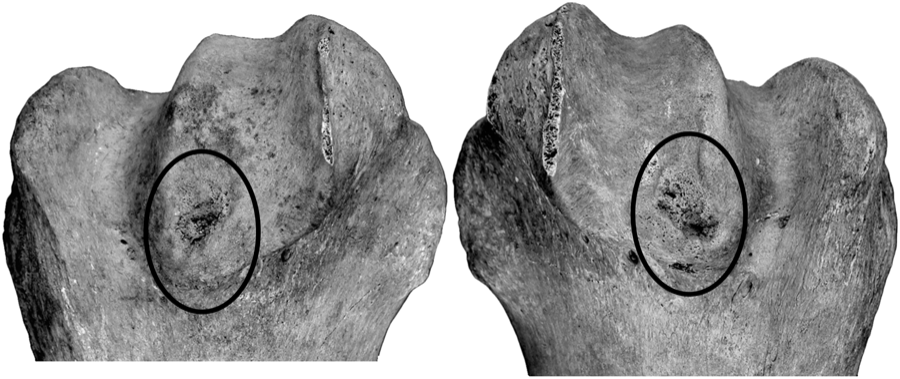
Symmetrical lesions at the distal end of the Tuberculum intermedium on left and right humeri.

The left mandible shows a minor malposition of the cheek tooth row as the mesial part of the P_3_ is rotated in labial direction. The upper canine shows densely packed circular grooves on the dentine of the root. The right mandible has periostitic bone depositions at the *margo ventralis* of the *diastema*. Although it can easily be detached it modified the mandibular bone surface there. Both scapulae show symmetrical periostitic bone deformations in the area of the *angulus caudalis* on its medial side (Fig. 8). Both humeri indicate symmetrical deformations at the distal end of the *tuberculum intermedium* where oval shaped lesions probably specify surface bone defects with traces of inflammations inside and around them (Fig. 9).

The anterior part of the *processus spinosi* in some cervical vertebrae is widened and show traces of inflammation on the surface. Several thoracic vertebrae have bent *processus spinosi*. Clear pathological changes, due to mechanical stress can be detected at the *autopodium* of the Tulln specimen. Especially the anterior coronal bones show severe palmar osteophytes and pathological bone growth at the palmar axial and abaxial corpus (Fig. 5). The posterior medial phalanges show pathologic alterations at the axial and abaxial margins of the corpus of the distal trochlea but not on their shafts (Fig. 6).

## Discussion

The identification of isolated archaeological faunal remains often entails difficulties and uncertain results. Although the Tulln specimen was preserved almost completely and subjected to intensive morphological and morphometrical examinations, the species identification turned out to be problematic due to its intermediate position between Bactrian camel and dromedary (Fig. 7). Today, the two domestic species exist in geographically different areas. The dromedary ranges from North Africa to Western Asia and Australia [29,30,31] while the Bactrian camel occurs in the Far East and Central Asia [32]. Different adaptations and ecotypes as well as different types of breeds cause variability of the habitus and size for various purposes, as riding animals, beasts of burden or for exploitation of meat and dairy products [29,33,34]. The sexual dimorphism also effects the discrimination of camel remains [35] as male dromedaries are about 10% heavier and 10 cm taller at shoulder height [36,37]. Depending on the animal’s age, castration certainly influences the skeleton and induces uncertainties in the identification of Bactrian camel *versus* dromedary bones. However, castrated camels are easier to handle and more effective as working animals and in meat production [34,38]. The cranial part of the pelvic symphysis certainly indicated a male Tulln specimen, but the smooth and weakly structured surface of the cranial part of the corpus pubis probably might hint to a castration.

The two species are able to interbreed, which results in larger, more powerful and efficient hybrid offspring [33,39,40,41]. Crossbreeding probably took first place in Assyria at the beginning of the 1st millennium BC [42] and this technique continued over Antiquity towards the modern times [4,34]. Hybridization was improved as the Arabs went into Iran and Central Asia [29,33]. Obviously, such hybrids were of great importance in the Ottoman troops mainly for transportation but also as riding animals [43,44,45]. The first appearance of a *camelry*, soldiers fighting with bows on camelback, had been recorded during the invasion of Xerxes in Greece 481 BC [39]. Today, hybridization facilitates improved milk and wool yield in hybrid *Tulu* or *Nar* camels from Middle Eastern and Central Asian countries. Commonly, two hybridizing methods are recognized, *Kurt-nar* (dromedary female x Bactrian male) and *Kez-nar* (Bactrian female x dromedary male) followed by F_1_-backcrossing with either dromedary for increased milk productivity or Bactrian camel for a higher wool yield and cold resistance [46,47]. A remarkable and relished sport is common in western Turkey, where highly valued male *Tulus* fight against each other in strictly regulated “camel wrestling” competitions (47, 48).

A lack of osteo-morphological characterization of hybrids [4,18,48] in literature and reference collections prevents a discrimination of hybrids and parental species. Nevertheless, the dromedary has more slender and the Bactrian camel broader long bones [17,40]. The long bones of the Bactrian camel are more robust while the dromedary has longer zygopodial and metapodial bones [32]. Most of the long bones of the Tulln specimen are rather long while their robustness addresses a Bactrian camel. Especially radius and femur have an intermediate position between both species (Fig. 7). Not so for the metacarpals, which are short and broad like in Bactrian camel (Fig. 7). The morphological features incorporate characteristics of both species, confirmed by the results of the nuclear DNA analyses as the Tulln-specimen turned out to be an F_1_ hybrid *Tulu* with a dromedary in its maternal and evidences of a Bactrian camel in its paternal line. We compared the detected mitochondrial haplotpye with a modern sequence data set of dromedaries throughout their distribution range. The hybrid camel from Tulln shares a main mitochondrial haplotype, which is common in nearly 70% of the modern dromedaries [49]. The results confirm previously described traditions that people in Anatolia imported Bactrian males to interbreed with Arabian female dromedaries but Bactrian females were and are of less interest [41,46].

Pathological deformities on the scapulae and the upper arm bones require special attention because they occur symmetrically. Probably, the *periostitis ossificans* at the mandible is caused by pressure of the harness. The periostitically changed bone surface medial on both shoulder blades perhaps originates in high strains on the *musculus serratus ventralis* that could be related to frequent coming down and going up of the individual to let the camel rider on and up (Fig. 8). The lesions under the exposed *tuberculum intermedium* (Fig. 9) are not easily explainable but might be related to stress-born deformities of the overlaying tendons of origin of the *musculus biceps brachii*. However, if the Tulln-specimen was used as a beast of burden, a higher frequency of stress related osteological changes should be expected. In this case only a few deformities mainly at the acropodium (Fig. 5, Fig. 6) and some bent dorsal spines of the thoracic vertebrae are proven. Such an ensemble of pathologies rather indicates a camel that was used as a riding animal and kept under good conditions.

It is impossible to reconstruct how the camel did arrive within the town walls of Tulln. Its appearance might be linked to an exchange of local people with the troops or the Ottoman army simply left it behind. Apparently, the citizens took it inside the town, where they probably kept and displayed it as an “exotic animal”. Further archeological research might answer this question. It seems quite conceivable that being not familiar with behavioral and feeding habits, the scarcity of food in wartimes, people did not keep it for long. As the camel died the citizens of Tulln did not exploit this alien animal, which the soldiers of the Ottoman troops certainly would have done. Exploitation of camel flesh especially in times of need was absolutely necessary. The dismemberment of the carcasses certainly is a reason for the scarce preservation of camel finds in general and is indicated by bones with butchering marks in particular. However, the citizens buried this camel in a typical post-mortal position, and together with rubbish in the remnants of a cellar that was leveled. The skeleton remained there for more than 300 years to raise questions in the future. Finally, it served as a perfect study example for a successful cooperation of the complementary fields of archaeological and genetic sciences. The camel specimen from Tulln is the first archaeozoologically and genetically confirmed evidence of a *Tulu* hybrid camel.

## Supporting Information

S1 Table Measurements in millimeter on long bones of the Tulln-specimen. (XLSX)

S2 Table Measurements in millimeter on the autopodium of the Tulln-specimen. (XLSX)

S3 Table Measurements in millimeter on vertebrae of the Tulln-specimen. (XLSX)

S4 Table mtDNA primer pairs used to amplify 532 bp fragment of control region. (PDF)
