# Supplementary material for: A Sunken Ship of the Desert at the River Danube in Tulln, Austria
Source: PLoS One. 2015 Apr 1;10(4):e0121235. doi: 10.1371/journal.pone.0121235 (PMC4382051; doi:10.1371/journal.pone.0121235)
Supplement: S4 Table — (PDF) [file pone.0121235.s004.pdf]

S4_Table.pdf: mtDNA primer pairs used to amplify 532 bp fragment of control region.

Primer ID	Sequence (5’ - 3’)	Tm ° C	Product Size (bp)
F01	RCCACACCCTCCCTAAGACT	60.51	92
R01	CGGAGGTCAGGGGGTAGT	59.91	
F02	CACCCAAAGCTGGAATTCTT	59.17	100
R02	GGCATGAYATGTGGTTTTTAG	58.01	
F03	ACGGCAATAGCCCTTGAGTA	59.73	97
R03	CAACGCGTGCTGTGACAT	60.5	
F04	GCGTRCATGAAACCTCAATA	59.69	90
R04	TATATGCATGGGGCAAACAA	59.78	
F05	TGTTTGCCCCATGCATATAA	59.78	85
R05	TGCGTATTGACTGGAAATGA	57.7	
F06	CRCATTATGTCAAATCATTTCC	59.33	99
R06	CTGCYRAGCGGGTTGATGAT	60.24	
F07	CCGCGTGAAATCATCAACC	62.41	94
R07	TGCCTGGTAAAGTTCCGGTAT	60.3	
F08	CATCCATTGTGGGGGTTTCT	61.9	86
R08	AGTGTGGGCGATTTTAGGTG	59.99	
F09	GGACCATCTCACCTAAAATCG	58.52	80
R09	GGCATGGGCTGATTAGTCATT	61.22	
F10	GGCATCTGGTTCTTACTTCAGG	60.13	100
R10	GGCATGGGCTGATTAGTCATT	61.22	
F11	CGGCATAACTGTGGTGTCAT	59.45	80
R11	AGACGGCCATAGCGGAGT	60.79	
F12	CTTGCAAGGACTCCGCTATG	60.92	98
R12	TGGTTGTATGATGCGGGTAA	59.81	
F13	CAAATCAATTGAAGCTGGACTTT	59.66	95
R13	TGTAGTTATGTCCTGCGACCA	59.21	
F14	CCGTCGCAGTCAAATCAA	59.33	99
R14	TATGTCCTGCGACCATTGAC	59.53	
